# The association between gut microbiome and hypertension varies according to enterotypes: a Korean study

**DOI:** 10.3389/frmbi.2023.1072059

**Published:** 2023-05-26

**Authors:** Ju Sun Song, Joung Ouk Ryan Kim, Sung Min Yoon, Min-Jung Kwon, Chang-Seok Ki

**Affiliations:** ^1^ Medical Office Unit, GC Genome, Yongin-si, Republic of Korea; ^2^ GC Labs, Department of Laboratory Medicine, Yongin-si, Republic of Korea; ^3^ Department of AI and Big Data, Swiss School of Management, Bellinzona, Switzerland; ^4^ Department of Laboratory Medicine, Kangbuk Samsung Hospital, Sungkyunkwan University School of Medicine, Seoul, Republic of Korea

**Keywords:** gut microbiome, hypertension, enterotype, *Faecalibacterium*, diet

## Abstract

**Introduction:**

Several animal and clinical studies have reported that the state of the human gut microbiome is associated with hypertension. In this study, we investigated the association between the gut microbiome and hypertension in a Korean population from an enterotypic perspective.

**Methods:**

A total of 623 participants were enrolled from a healthcare center and classified into four enterotypes, *Bacteroides*1- (*Bac*1), *Bacteroides*2- (*Bac*2), *Prevotella*- (*Pre*), and *Ruminococcus* enterotype-like-composition (*Rum*).

**Results:**

When comparing the four enterotypes, clinical characteristics related to obesity, metabolic syndrome, and blood pressure were significantly associated with th e enterotypes, showing unfavorable associations with the *Bac*2 group and the opposite for the *Rum* group. Similarly, the prevalence of hypertension was highest in the *Bac*2 group and lowest in the *Rum* group. When analyzing the association between gut microbiota and blood pressure for each enterotype, gut microbial features of lower diversity, depletion of important short chain fatty acid-producing taxa, such as *Faecalibacterium*, *Blautia*, *Anaerostipes*, and enrichment of lipopolysaccharide -producing taxa, such as *Megamonas*, were found only in the dysbiotic *Bac*2 group.

**Discussion:**

From an enterotype perspective, this study on a large Korean cohort shows that low-diversity *Bacteroides*2-enterotype-like composition is associated with hypertension, while the reverse is true for high-diversity *Ruminococcus*-enterotype-like composition and, to a limited degree, *Bacteroides*1-enterotype-like composition. In addition, we suggest that the effect of gut microbiota-mediated risk of hypertension could be modulated by altering the gut microbiome *via* diet. Dietary intervention trials promoting a balanced Korean diet instead of a more Western alternative may provide more definitive evidence for the involvement and role of the gut microbiome in relation to blood pressure.

## Introduction

Hypertension is a major global health issue affecting 1.13 billion people worldwide, according to the World Health Organization. It increases the risk of cardiovascular diseases, including heart disease and stroke, and represents a tremendous public health burden. Its high prevalence is also an issue in Korea, with the number of patients with hypertension exceeding 12 million in 2018 (Kim et al., 2021) and continuously increasing, owing to the rapid aging of the population and westernized lifestyles.

Hypertension is a complex multifactorial disease, with genetic, environmental, and demographic factors contributing to its prevalence. Recently, animal studies have indicated that the gut microbiota play an important role in the regulation of blood pressure (Yang et al., 2015; Adnan et al., 2017; Santisteban et al., 2017). Numerous clinical studies have attempted to elucidate the relationship between the gut microbiota and hypertension, as well as to identify microbial markers of hypertension (Li et al., 2017; Yan et al., 2017; Sun et al., 2019; Nagase et al., 2020; Verhaar et al., 2020; Maifeld et al., 2021; Nakai et al., 2021). Metabolites produced by the gut microbiota, such as short-chain fatty acids (SCFAs), trimethylamine *N*-oxide (TMAO), and lipopolysaccharides (LPS), are suggested to directly affect endothelial, kidney, and heart tissues, amongst others (Al Khodor et al., 2017; Kang and Cai, 2018; Marques et al., 2018; Oyama and Node, 2019; Yang et al., 2020). However, even large-cohort studies have not identified consistent microbial markers and occasionally produce slightly conflicting results. Therefore, the relationship between hypertension and the gut microbiota still needs to be elucidated (Sun et al., 2019; Palmu et al., 2020; Verhaar et al., 2020).

The gut microbiome exhibits large individual differences and is affected by various factors. Among them, diet has a significant influence on the human gut microbiota. Variations in the gut microbiota exist regardless of age, sex, ethnicity, and geography, and are mainly determined by habitual diet. These recurrent patterns of microbial composition in the gut microbiome can be separated into several clusters termed the “enterotype.” These enterotypes are functionally and ecologically different. Therefore, it can be assumed that different enterotypes and their microbial architectures influence the development of hypertension in different ways and to varying degrees.

To elucidate these interactions and investigate the microbial markers associated with hypertension, in this study, we analyzed the association between the gut microbiota and hypertension across different enterotypes using 16S rRNA amplicon sequencing data.

## Materials and methods

### Study participants

Individuals aged 19 years or older who underwent health checkups were recruited from the clinics of the Kangbuk Samsung Hospital Total Healthcare Centers in Seoul and Suwon, South Korea. Demographic and clinical data were collected from health checkup reports. Patients’ blood pressure was measured three times, and the average was considered as the final measurement. According to the 2017 guidelines for high blood pressure in adults (Whelton et al., 2017), participants with systolic blood pressure (SBP) < 120 mmHg and diastolic blood pressure (DBP) < 80 mmHg were classified into the normotension group, whereas participants with SBP > 130 mmHg or DBP > 80 mmHg were classified into the hypertension group. Patients with cancer, active intestinal inflammatory diseases, renal failure, heart failure, peripheral artery disease, or secondary hypertension were excluded from the study. None of the participants received antihypertensive treatment. Individuals receiving antibiotics or probiotics during the preceding three months were also excluded.

This study was approved by the Ethics Committee of the Gangbuk Samsung Hospital (protocol number: KBSMC 2019-04-040), and all participants provided written informed consent.

### Fecal sample collection and 16S rRNA sequencing

Fecal samples were self-collected using a stool collection kit (NBgene-GUT kit; Noble Biosciences, Republic of Korea) containing preservatives. All samples arrived within 3 days of sampling. DNA was immediately extracted upon arrival of the samples using a Chemagic DNA Stool Kit (PerkinElmer, USA) with a modified bead-beating pretreatment step. Each sample was aliquoted into a bead tube (Lysing matrix E; MP Biomedical, USA) and homogenized using a Fastprep-24 homogenizer for 1 min. The V4 hypervariable region was amplified using a NEXTflex 16S V4 Amplicon-Seq kit (BioO Scientific, Austin, TX, USA) and sequenced using an Illumina MiSeq Reagent Kit v2 (500 cycles) following the manufacturer’s protocol.

### Metagenomic analyses of faecal samples

At least 20,000 reads were obtained per sample and sequence reads were analyzed using the QIIME 2 framework (Bolyen et al., 2019). Demultiplexed and primer-trimmed data were quality-filtered and denoised using the DADA2 plugin (Callahan et al., 2016). Amplicon sequence variants (ASV) with fewer than 10 reads or those present in only a single sample were removed, and each amplicon sequence variant was assigned using naive Bayes machine-learning taxonomy classifiers in the q2-feature-classifier (Bokulich et al., 2018) trained against the NCBI refseq database. The data were transformed into proportions by dividing the number of reads for each taxon in a sample by the total number of reads in that sample. Finally, rare taxa with an abundance of less than 0.1% were removed.

### Statistical analysis

The Shannon diversity index was calculated to determine alpha diversity, and the Bray–Curtis dissimilarity on the genus-level relative abundance matrix was used to compare the communities. A linear discriminant analysis effect size (LEfSe) approach was adopted to discover microbiological markers associated with hypertension status using the default settings (e.g., inear discriminative analysis [LDA] score >2) (Segata et al., 2011). Enterotyping of the genus-level abundance microbial profiles was performed using the Dirichlet multinomial mixtures (DMM) approach implemented in the R package DirichletMultinomial (Holmes et al., 2012). Inter-individual microbiome variation was visualized using principal coordinate analysis. PERMANOVA using the “adonis” command in the vegan package of R (10,000 simulations) (Warton et al., 2012) was used for microbial community comparisons.

The explanatory power of clinical variables and their effect size on microbial community variation were evaluated using distance-based redundancy analysis (db-RDA) performed at the genus level using the Bray–Curtis dissimilarity matrix, as implemented in vegan (Oksanen et al., 2014).

Statistical comparisons of clinical variables and alpha-diversity of the gut microbiota were performed using the Mann–Whitney U-test and Kruskal–Wallis test, with a post-hoc Dunn test for two groups and more than three groups, respectively. Statistical differences in categorical variables and the prevalence of enterotypes between the groups were evaluated using the chi-square test and pairwise Fisher’s exact tests, respectively.

## Results

### Study participants and comparison of clinical and gut microbial features between normotension and hypertension groups

**Figure 1:**
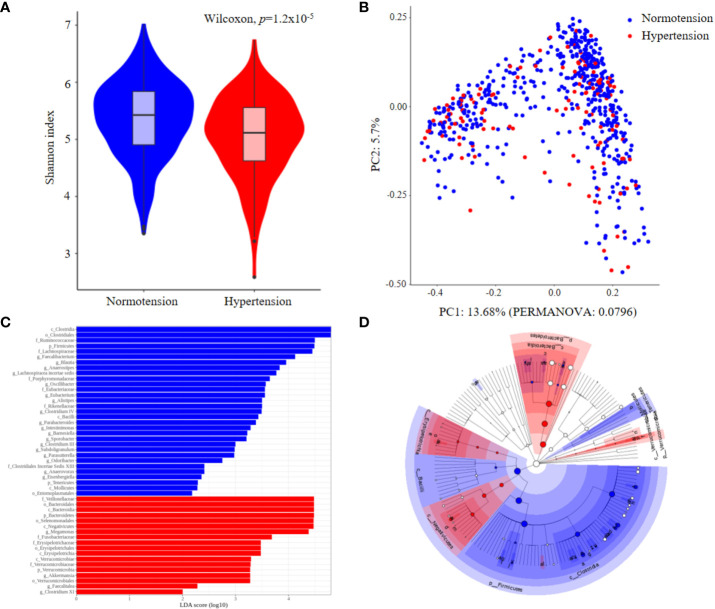
Comparison of gut microbial features between normotension and hypertension groups for the entire cohort. **(A)** Alpha-diversity. **(B)** Principal coordinates analysis (PCoA) derived from Bray–Curtis distances. **(C)** Linear discriminative analysis (LDA) effect size (LEfSe) analysis. **(D)** Cladogram showing differentially abundant taxonomic clades with an LDA score >2.0.

We enrolled 623 participants, including 503 normotensive individuals and 120 patients with hypertension; Their clinical metadata and ASV data for 16S rRNA sequencing are presented in **Supplementary Table 1**, their clinical characteristics are listed in **Table 1**. BMI and waist circumference showed the most significant differences between the two groups, followed by sex, triglyceride level, and age. While the beta diversity, as calculated by the Bray-Curtis distance, did not differ between the hypertension and normotension groups (*p*=0.0796, **Figure 1B**), the alpha diversity of the gut microbiota for the hypertension group was significantly lower than that for the normotension group (*p*=1.2x10^-5^, **Figure 1A**). By conducting a LEfSe analysis across all taxa, from phylum to genus, we identified distinct features that were differentially abundant between the two groups. **Figures 1C, D** show that the normotension group exhibited higher levels of Gram-positive bacteria, such as *Clostridia*, *Ruminococcaceae*, and *Lachnospiraceae*, which are mostly SCFA-producing bacteria. Conversely, the hypertensive group had higher levels of Gram-negative bacteria, predominantly from the families *Bacteroidetes* and *Negativicutes*.

**Table 1:** Demographic and clinical characteristics of the total cohort of participants.

Characteristics	Normotension (n=503)	Hypertension (n=120)	*p*-value
SBP, mmHg	104.6 (8.3)	126.3 (9.5)	3.8 x 10^-58^
DBP, mmHg	66.8 (6.4)	86.2 (5.4)	2.3 x 10^-64^
Age, y	42.3 (8.1)	45.1 (6.9)	4.3 x 10^-05^
Sex (Male)	208 (41.4)	86 (71.7)	2.3 x 10^-09^
BMI, kg/m^2^	23.1 (3.5)	25.4 (3.0)	2.7 x 10^-13^
Waist circumference, cm	79.4 (9.4)	86.8 (7.5)	2.8 x 10^-16^
T-chol, mg/dL	192.0 (32.6)	199.7 (32.1)	0.017
LDL, mg/dL	126.3 (30.8)	132.0 (32.7)	0.043
HDL, mg/dL	62.5 (15.6)	58.1 (15.2)	0.0037
Triglyceride, mg/dL	102.4 (62.1)	142.4 (88.5)	1.3 x 10^-8^

Data are displayed as means and (SD) for continuous values and n (%) for dichotomous values. P-values: Mann-Whitney test for continuous variables, Chi-squared test for categorical variables.

### Enterotype clustering

**Figure 2:**
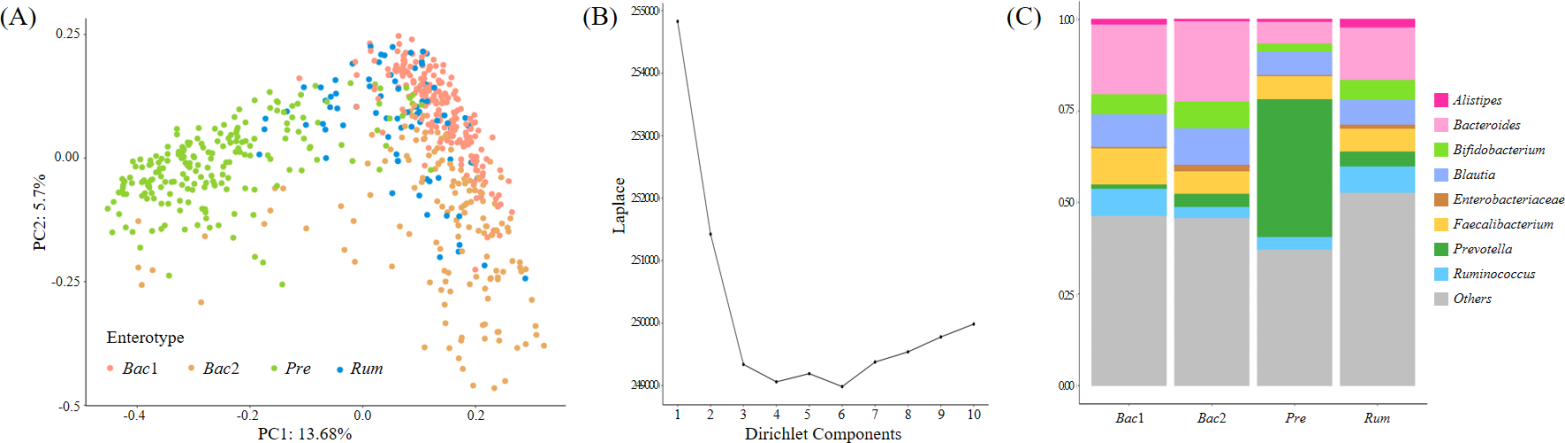
Enterotyping of the entire cohort. **(A)** Principal coordinate visualization of the four enterotypes resulting from community typing, performed using the Dirichlet Multinomial Mixture Model (DMM) on genus-level gut microbiome profiles. **(B)** LaPlace approximation of DMM Model fit supporting the recognition of four distinct microbiota structures in our cohort. **(C)** Stacked bar graphs indicating that each DMM cluster is dominated by a distinct bacterial genus.

We enterotyped the entire cohort using Dirichlet multinomial mixtures on genus-level profiles to explore the potential relationship between the fecal microbiome community constellations and clinical characteristics, particularly blood pressure. The LaPlace approximation of the DMM Model fit determined the number of optimal clusters that best fit the data, and four distinct microbiota were distinguished (**Figures 2A, B**). These four enterotypes were designated according to their relative abundance profiles as follows: *Bacteroides*1*-* (*Bac*1), *Bacteroides*2*-* (*Bac*2), *Prevotella-* (*Pre*), and *Ruminococcus*-enterotype-like composition (*Rum*), as depicted in **Figure 2C**. A total of 201, 144, 201, and 77 individuals were classified as *Bac*1, *Bac*2, *Pre* and *Rum*, respectively.

### Comparison between different enterotypes

We compared the clinical characteristics (**Table 2**) and gut microbial features of the four enterotypes. Five of the clinical variables, i.e., BMI, waist circumference, triglyceride, SBP, and DBP, showed the most significant differences and were all related to obesity or metabolic syndrome. The db-RDA showed the significant relevance of these five clinical variables in explaining the patterns of the four enterotypes (*p*<0.001 in univariate db-RDA), with 51.7% of the non-redundant cumulative explanatory power (**Supplementary Figure 1**).

**Table 2:** Each enterotype’s demographic and clinical characteristics.

Characteristics	*Bac*1 (n=201)	*Bac*2 (n=144)	*Pre* (n=201)	*Rum* (n=77)	*P*-value
SBP, mmHg	106.9 (11.7)	112.3 (12.9)	110.3 (11.7)	103.1 (9.6)	8.4 x 10^-8^
DBP, mmHg	69.1 (10.0)	73.5 (10.2)	71.3 (9.6)	67.0 (7.4)	2.6 x 10^-6^
Age, y	43.2 (7.5)	42.3 (8.4)	43.7 (8.3)	40.8 (6.5)	0.0381
Sex (Male)	65 (32.3)	84 (58.3)	123 (61.2)	22 (28.6)	0.0779
BMI, kg/m^2^	23.2 (3.5)	24.3 (3.8)	23.7 (3.3)	22.5 (3.0)	4.0 x 10^-4^
Waist circumference, cm	79.4 (9.6)	83.1 (10.2)	81.5 (8.8)	78.5 (8.8)	2.0 x 10^-4^
T-chol, mg/dL	193.5 (33.6)	195.9 (31.3)	192.2 (31.5)	192.6 (36.1)	0.7146
LDL, mg/dL	128.8 (32.7)	129.0 (29.8)	125.8 (30.1)	125.1 (33.6)	0.5747
HDL, mg/dL	62.0 (15.1)	60.5 (15.6)	60.8 (15.9)	65.2 (15.6)	0.1277
Triglyceride, mg/dL	102.1 (55.9)	128.1 (85.7)	112.2 (73.0)	91.7 (51.9)	0.001

Data are displayed as means and (SD) for continuous values and n (%) for dichotomous values. P-values: Mann-Whitney U test for continuous variables, Cochran-Mantel-Haenszel test for categorical variables.

Enterotype-like compositions: Bac1, Bacteroides1; Bac2, Bacteroides2; Pre, Prevotella; Rum, Ruminococcus.

**Figure 3:**
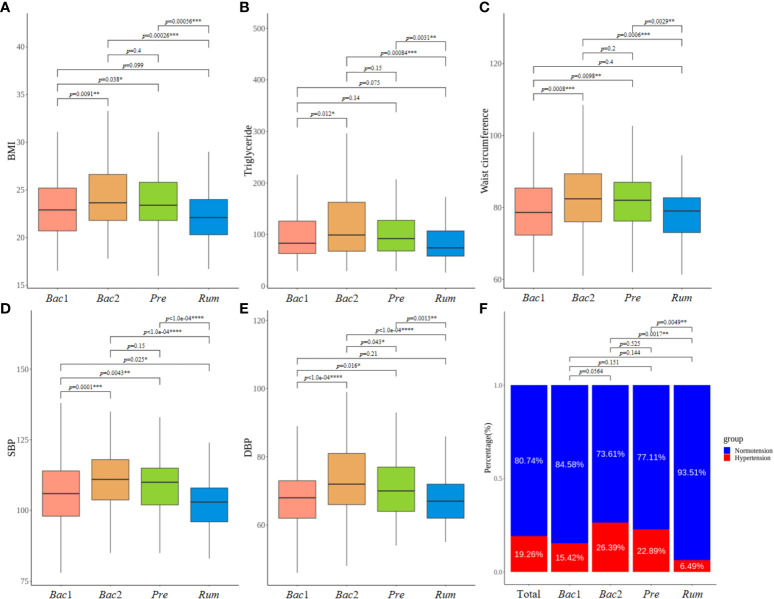
Comparison of clinical characteristics between enterotypes. **(A–E)** Comparison of five clinical characteristics with most significant differences across four enterotypes. All of these variables, including blood pressure values, exhibited a consistent pattern of both elevation and reduction. **(B)** Prevalence of hypertension varied according to the enterotypes, showing the highest prevalence in the *Bact2* enterotype and the remarkable lowest prevalence in the *Rum* enterotype. *P*-values less than 0.05, 0.01, 0.001 and 0.0001 were marked with *, **, *** and ****, respectively.

Notably, individuals belonging to the *Bac*2 group displayed significantly higher values for these five clinical variables compared to individuals classified into the *Rum* and *Bac*1 groups, although there were no significant differences between the *Bac*2 and *Pre* groups. The *Rum* group showed the lowest values for the clinical variables (**Figures 3A–E**). The five aforementioned clinical variables, encompassing blood pressure, exhibited consistent patterns of elevation and reduction. The *Bac*2 group had the highest prevalence of hypertension (26.4%), followed by the *Pre* (22.9%), *Bac*1 (15.4%), and *Rum* groups (6.5%). The prevalence of hypertension differed significantly between the *Bac*2 and *Rum* groups (pairwise Fisher’s exact test, *p*=0.002), and the *Pre* and *Rum* groups (*p*=0.005) (**Figure 3F**).

**Figure 4:**
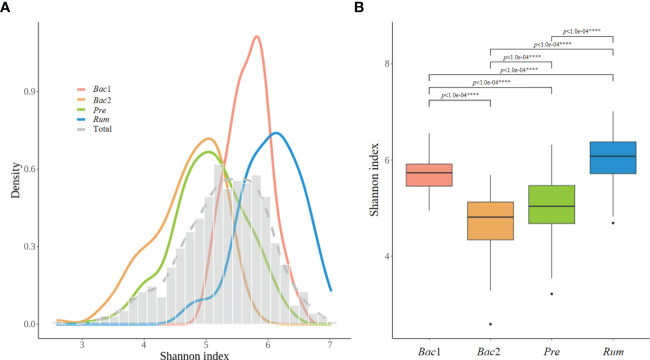
Alpha-diversity of the four enterotypes. **(A)** Distribution of alpha-diversity between enterotypes, with low diversity corresponding to the *Bact2* enterotype and high diversity corresponding to the the *Rum* enterotype. **(B)** Box plot representing the first and third quartiles of the distribution of alpha-diversity. The alpha diversity showed significant differences between all each other enterotypes. P-values less than 0.0001 was marked with ****.

The alpha diversity of the gut microbiome was assessed across enterotypes. Although the analysis of the prevalence of enterotypes along the alpha-diversity axis revealed a bimodal distribution consistent with previous observations, the location of the distribution differed between enterotypes, revealing the lowest and highest diversity in the *Bac*2 and *Rum* groups, respectively (**Figure 4A**). Additionally, the alpha diversity was significantly different across all enterotypes (Kruskal-Wallis test, *p*<0.0001) (**Figure 4B**). The diversity of the gut microbiota exhibited a positive correlation with the above five clinical characteristics, and these findings provide evidence for the dysbiotic microbiotal configurations associated with the *Bac*2 group.

### Comparison of normotension and hypertension groups for each enterotype

We then compared the normotension and hypertension groups for each enterotype. The clinical characteristics of the patients are shown in **Table 3**. BMI, waist circumference, and age were usually different between the two blood pressure groups for all enterotypes. However, high-density lipoprotein cholesterol (HDL) and triglyceride (TG) levels between the two blood pressure groups were significantly different only in the *Bac*1 group (**Table 3**).

**Table 3:** Comparison of demographic and clinical characteristics between normotension and hypertension groups in each enterotype.

Characteristics	*Bac*1		*Bac*2		*Pre*		*Rum*	
	NL (n=170)	HT (n=31)	NL (n=106)	HT (n=38)	NL (n=155)	HT (n=46)	NL (n=72)	HT (n=5)
SBP, mmHg	103.4 (8.7)	125.6 (7.5)	106.7 (7.8)	127.8 (11.7)	105.6 (7.6)	126.0 (9.4)	101.8 (8.5)	121.8 (1.9)
DBP, mmHg	65.8 (6.8)	86.9 (4.5)	68.6 (6.1)	87.3 (5.9)	67.2 (6.1)	85.1 (5.6)	65.9 (6.3)	83.0 (2.2)
Age, y	42.9 (7.7)	45.3 (5.9)	41.6 (9.0)	44.2 (6.2)	43.1 (8.3)	45.5 (8.2)	40.5 (6.5)	46.2 (6.0)
Sex (Male)	50 (29.4)	15 (48.4)	**55 (51.9)**	**29 (76.3)**	**84 (54.2)**	**39 (84.8)**	19 (26.4)	3 (60.0)
BMI, kg/m^2^	**22.7 (3.4)**	**25.9 (2.8)**	**23.7 (3.7)**	**26.0 (3.6)**	23.5 (3.5)	24.6 (2.4)	22.3 (3.0)	25.3 (2.5)
Waist circumference, cm	**78.0 (9.3)**	**87.5 (6.5)**	**80.9 (9.8)**	**89.1 (8.7)**	80.7 (9.2)	84.2 (6.6)	**77.8 (8.5)**	**88.3 (7.9)**
T-chol, mg/dL	191.6 (33.2)	203.6 (34.5)	195.9 (30.7)	195.9 (33.3)	189.9 (31.4)	199.8 (30.9)	191.8 (36.7)	203.2 (25.0)
LDL, mg/dL	126.4 (31.8)	141.9 (34.7)	130.2 (28.8)	125.6 (32.5)	124.2 (29.4)	131.1 (32.1)	125.0 (34.4)	126.6 (23.3)
HDL, mg/dL	**63.7 (14.9)**	**52.7 (12.9)**	61.0 (16.3)	59.3 (13.8)	61.1 (16.3)	59.8 (14.9)	65.1 (14.1)	66.4 (33.0)
Triglyceride, mg/dL	**94.4 (50.6)**	**144.0 (65.5)**	117.2 (69.7)	158.3 (115.6)	107.7 (70.4)	127.7 (80.2)	88.0 (50.6)	146.2 (41.5)

NL, normotension; HT, hypertension.

* Data are displayed as means and (SD) for continuous values and n (%) for dichotomous values. P-values: Mann-Whitney test for continuous variables, Chi-squared test for categorical variables. Statistically significant values (p<0.01 are indicated in bold).

Enterotype-like compositions: Bac1, Bacteroides1; Bac2, Bacteroides2; Pre, Prevotella; Rum, Ruminococcus.

**Figure 5:**
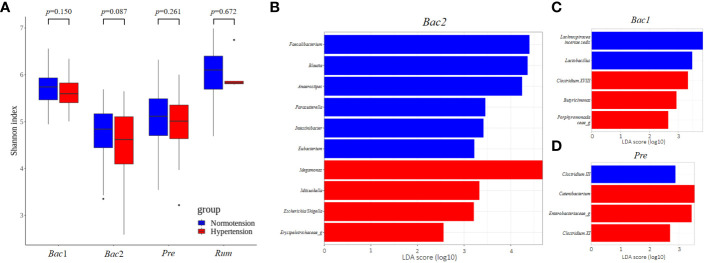
Comparison of gut microbial features between two blood pressure groups in each enterotype. **(A)** Comparison of alpha-diversity. Of the four enterotypes, only the *Bact2* enterotype showed a significantly lower diversity in the hypertension group. **(B–D)** Linear Discriminant Analysis Effect Size (LEfSe) analysis, showing those genera with significantly different abundances between the two blood pressure groups. There were no significantly different taxa in the *Rum* enterotype. Significantly different taxa with an LDA score greater than 4 were only found in the *Bact2* enterotype.

**Figure 6:**
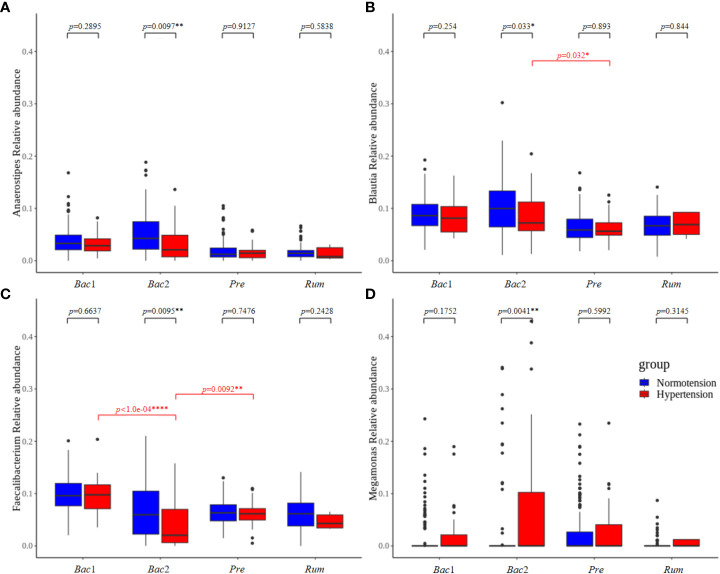
The taxa that showed significant differences between the normotension and hypertension groups belonged to the *Bact2* enterotype. *Faecalibacterium*, *Blautia*, *Anaerostipes*, and *Megamonas* were compared in the entire cohort. Of the hypertension groups, only *Faecalibacterium* belonging to the *Bact2* enterotype was significantly lower than the other enterotypes; the other taxa, *Blautia*, *Anaerostipes*, and *Megamonas*, did not show any significant differences between the hypertension groups of four enterotypes. P-values less than 0.05, 0.01, and 0.0001 were marked with *, **, and ****, respectively.

To evaluate the association between gut microbial features and hypertension with respect to enterotype, we compared the gut microbial features of the normotension and hypertension groups for each enterotype. Notably, the other groups, with the exception of the *Bac*2 group, did not show any significant differences in alpha diversity between the two blood pressure groups, but the *Bac*2 group had slightly lower diversity in the hypertension group (Kruskal-Wallis, *p*=0.087) (**Figure 5A**). In the analysis of beta diversity, no enterotypes exhibited statistically significant differences between the two blood pressure groups (**Supplementary Figure 2**). To investigate the distinctive taxa between the two blood pressure groups for each enterotype, we conducted a LEfSe analysis at the genus level. Except for the *Rum* group, all other groups revealed significant differences in taxonomic composition between the two blood pressure groups (**Figures 5B–D**). Among the taxa, genera with an LDA score greater than 4 were only detected in the *Bac*2 group, with depletion of *Faecalibacterium*, *Blautia*, and *Anaerostipes* and enrichment of *Megamonas* in the hypertension group. We compared these taxa in the entire cohort and found that among the hypertension groups *Faecalibacterium* in the *Bac*2 group was significantly lower than that in the *Bac*1 and *Pre* groups (Kruskal-Wallis, *p*<0.01), but the other taxa, *Blautia*, *Anaerostipes*, and *Megamonas*, did not show such differences between the four enterotypes (**Figure 6**).

## Discussion

In the overall comparison of gut microbial features between the normotension and hypertension groups, the latter was associated with low gut microbial diversity and some distinctive taxa, depleted SCFA-producing bacteria, such as *Ruminococcacea* and *Faecalibacterium*, and enriched LPS-producing gram-negative bacteria, such as *Bacteroidales*, *Negativiticus*, and *Megamonas*, although there were no significant differences in gut microbial composition. SCFAs are the products of indigestible dietary fiber fermentation by colonic microbes and may alter blood pressure (Marques et al., 2018; Yang et al., 2020). They can directly regulate blood pressure by binding to the SCFA receptor on vascular smooth muscle and endothelial cells. Several animal studies have demonstrated a relationship between hypotensive effects and increased SCFA levels due to high-fiber diet intake, as well as acetate and propionate supplementation (Pluznick et al., 2013; Marques et al., 2017). Bacterial LPS is a representative pathogen-associated molecular pattern that allows human cells to detect bacterial invasion and initiate innate immune response (Matsuura, 2013). Colonic-derived LPS can pass into the circulatory system, thereby increasing the plasma LPS level (termed metabolic endotoxemia) and promoting systemic inflammation involved in various metabolic diseases, such as obesity, diabetes, and non-alcoholic fatty liver disease (Mohammad and Thiemermann, 2020).

In this study, we attempted to analyze the association between gut microbiota and hypertension in terms of enterotype, beyond simply comparing the gut microbiota of normotensive and hypertensive groups, because the microbial architecture is functionally and ecologically different between enterotypes. Enterotypes have generally been defined by the dominance of either *Bacteroides*, *Prevotella*, or *Ruminococcacea* (Arumugam et al., 2011) and are strongly associated with long-term diets. Individuals consuming carbohydrate-rich diets, including dietary fiber and simple sugars, belong to the *Prevotella* enterotype, whereas those consuming protein-and animal fat-rich diets belong to the *Bacteroides* enterotype (Wu et al., 2011). *Ruminococcaceae* is associated with the long-term consumption of fruits and vegetables (Tomova et al., 2019). Considering Korean diets, the *Ruminococcaceae* enterotype has been linked to adults on a Korean-style balanced diet (Wu X. et al., 2021). Recently, the splitting of the *Bacteroides* group into two subgroups, *Bacteroides* 1 and *Bacteroides* 2, showed that the latter could be linked to lower gut microbial gene richness and clinical characteristics, including severe obesity and systemic inflammation (Aron-Wisnewsky et al., 2019; Vieira-Silva et al., 2020). Our analysis also produced four enterotype clusters, in line with previous reports on gut microbiome community variation (Ding and Schloss, 2014; Vandeputte et al., 2017; Vieira-Silva et al., 2019).

Considering the clinical characteristics among the four enterotypes, the *Bac2* enterotype was associated with significantly higher levels of obesity or metabolic syndrome-defining variables, including BMI, waist circumference, triglyceride level, and blood pressure. In addition, the *Bac*2 enterotype was characterized by lower gut microbial diversity and decreased levels of *Faecalibacterium*, a potent butyrate-producing bacterium. These findings are consistent with those of most investigators, who suggested that the *Bacteroides2* enterotype is a dysbiotic gut microbiome with pro-inflammatory properties and is associated with obesity and inflammation-related diseases. In contrast, the *Rum* enterotype in our study had the lowest values for these hypertension-related clinical characteristics, as well as the highest gut microbial diversity. The *Ruminococcaceae* enterotype has been considered abundant for the gut microbe producing anti-inflammatory compounds, causing a lower inflammatory response (Tomova et al., 2019; Alili et al., 2021; Brial et al., 2021; Wu X. et al., 2021; Geng et al., 2022).

Therefore, the gut microbial features identified as being related to blood pressure in the entire cohort, such as gut microbial diversity and relative abundance of SCFA- or LPS-producing bacteria, seemed to be the features associated with the *Bac2* or *Rum* enterotypes rather than those directly related to blood pressure.

We tested the association between gut microbiota and blood pressure for each enterotype to clarify the presence of an enterotype-mediated gut microbial risk pattern determined by the local microenvironment. The results revealed that, with the exception of the *Bac2* enterotype, there were no associations between hypertension status and gut microbiota in the *Pre*, *Bac1*, and *Rum* enterotypes. However, the clinical characteristics significantly associated with hypertension in the entire cohort showed significant differences among these three enterotypes. Therefore, the development of hypertension in these enterotypes could be attributed to typical clinical features related to hypertension, such as aging, sex, obesity, or lipid value, rather than the gut microbiota. Interestingly, HDL and TG levels only showed a significant association with hypertension in the *Bac1* enterotype. Despite the lack of a precise understanding of the underlying mechanism, it is believed to be related to the animal food-based dietary habits of the *Bacteroides* enterotype.

Interestingly, only in the *Bac2* enterotype, there were differences in the gut microbial features such as the microbial diversity and relative abundance of taxa including *Faecalibacterium*, *Blautia*, *Anaerostipes*, and *Megamonas*, as well as in the clinical characteristics related to hypertension between the two blood pressure groups. However, among those distinctive taxa, only *Faecalibacterium*, but not *Blautia*, *Anaerostipes*, or *Megamonas*, was significantly lower in the hypertension group of the *Bac2* enterotype than those in the other enterotypes. Although the *Bac2* enterotype inherently possesses dysbiotic traits, the observation that the *Faecalibacterium* proportion of the gut microbiome in the *Bac2* enterotype hypertension group was significantly lower than that in the hypertension groups of the other enterotypes implies that hypertension in this particular enterotype is additionally linked to gut microbiome dysbiosis, as well as clinical manifestations.

In particular, butyrate can enter the bloodstream and exert a potent hypotensive effect by preventing vascular inflammation. It can also act on vagal afferent neurons and the central nervous system to affect blood pressure (Onyszkiewicz et al., 2019; Wu Y. et al., 2021). Consistent with previous results, *Faecalibacterium* which is considered the most potent butyrate-producing bacteria and the biomarker most closely associated with hypertension prevention also revealed to had the strongest association with decreased blood pressure in our study.

We discovered that the clinical phenotypes associated with obesity or metabolic syndrome, including hypertension, showed an unfavorable association with the *Bac2* enterotype and a protective relationship with the *Rum* enterotype. Considering this result, the increasing number of people with hypertension in Korea (Kim et al., 2021) may be related to the increasing prevalence of the *Bacteroides* enterotype, as well as the loss of microbial diversity and SCFA-producing bacteria in the gut, due to the westernization of diet in the Korean population. Therefore, our findings are consistent with previous reports of lowered cardiometabolic risk profiles in participants consuming diets rich in fruits and vegetables (Borgi et al., 2016).

Furthermore, we found that among the four enterotypes the pro-inflammatory features of depleted SCFA-producing bacteria were associated with hypertension in the dysbiotic *Bac2* enterotype, and that the effect of gut microbiota-mediated risk for hypertension might be modulated according to the local microbial environment. Many human intervention studies aimed at reducing blood pressure through the modulation of the gut microbiome using dietary fiber, prebiotics, or postbiotics are ongoing. However, considering our study’s results, it seems that the impact of these interventions may differ depending on the enterotype. Clinical trials with stratification of the target population according to enterotype, and those comparing the effectiveness of SCFAs in reducing blood pressure across different enterotypes, may provide a reference for creating treatment guidelines that screen and select the population in line with microbial modulation as the primary treatment, thus opening up the possibility of a tailored approach in the treatment of hypertension.

To our knowledge, this is the first study to evaluate the association between the gut microbiota and hypertension in a large Korean cohort. This study assessed the enterotype-based relationship between the gut microbiome and hypertension and showed that low-diversity *Bacteroides*2-enterotype-like composition is associated with hypertension, while the reverse is true for high-diversity *Ruminococcus*-enterotype-like composition. As well as, the depletion of SCFA-producing bacteria and increase in LPS-producing bacteria as dysbiosis associated with hypertension were significant only in the *Bac*2 enterotype. Further prospective studies with larger sample sizes or other ethnicities could provide more definitive and significant evidence for assessing the involvement of enterotypes in the association between the gut microbiome and hypertension.

## Data availability statement

The datasets presented in this study can be found in online repositories. The names of the repository/repositories and accession number(s) can be found below: https://www.ebi.ac.uk/ena, PRJEB56540.

## Ethics statement

The studies involving human participants were reviewed and approved by Ethics Committee of Gangbuk Samsung Hospital. The patients/participants provided their written informed consent to participate in this study.

## Author contributions

JS conceived of the presented idea, planned the experiments and wrote the manuscript. JK and SY developed the theory and performed the computations. M-JK contributed to sample preparation. C-SK supervised the project. All authors contributed to the article and approved the submitted version.

## Supplementary material

Supplementary Table 1 Metadata and ASV data for each of the samples

Supplementary Figure 1 Clinical covariates correlating to microbiome community variation (dbRDA, genus-level Bray–Curtis distance), either independently (univariate effect sizes in black) or in a multivariate model (cumulative effect sizes in grey).

Supplementary Figure 2 Beta diversity as a principal coordinates analysis (PCoA) plot derived from Bray–Curtis distances of two blood pressure groups in each enterotype. There was no significant difference between the two groups for all four enterotypes. **(A)** *Bac*1 enterotype **(B)** *Bac*2 enterotype **(C)** *Pre* enterotype **(D)** *Rum* enterotype
