# Supplementary figures and images for: The association between gut microbiome and hypertension varies according to enterotypes: a Korean study
Source: Front Microbiomes. 2023 May 26;2:1072059. doi: 10.3389/frmbi.2023.1072059 (PMC12993491; doi:10.3389/frmbi.2023.1072059)

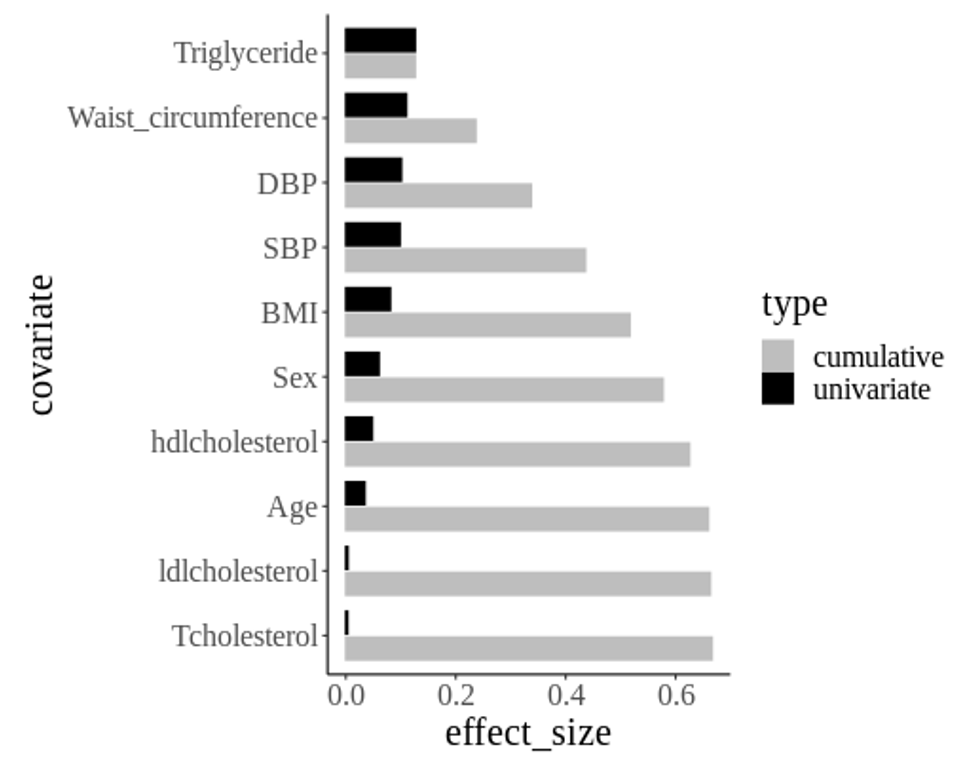

Supplement: Supplementary Figure 1 — Clinical covariates correlating to microbiome community variation (dbRDA, genus-level Bray–Curtis distance), either independently (univariate effect sizes in black) or in a multivariate model (cumulative effect sizes in grey). [file Image_1.tif]

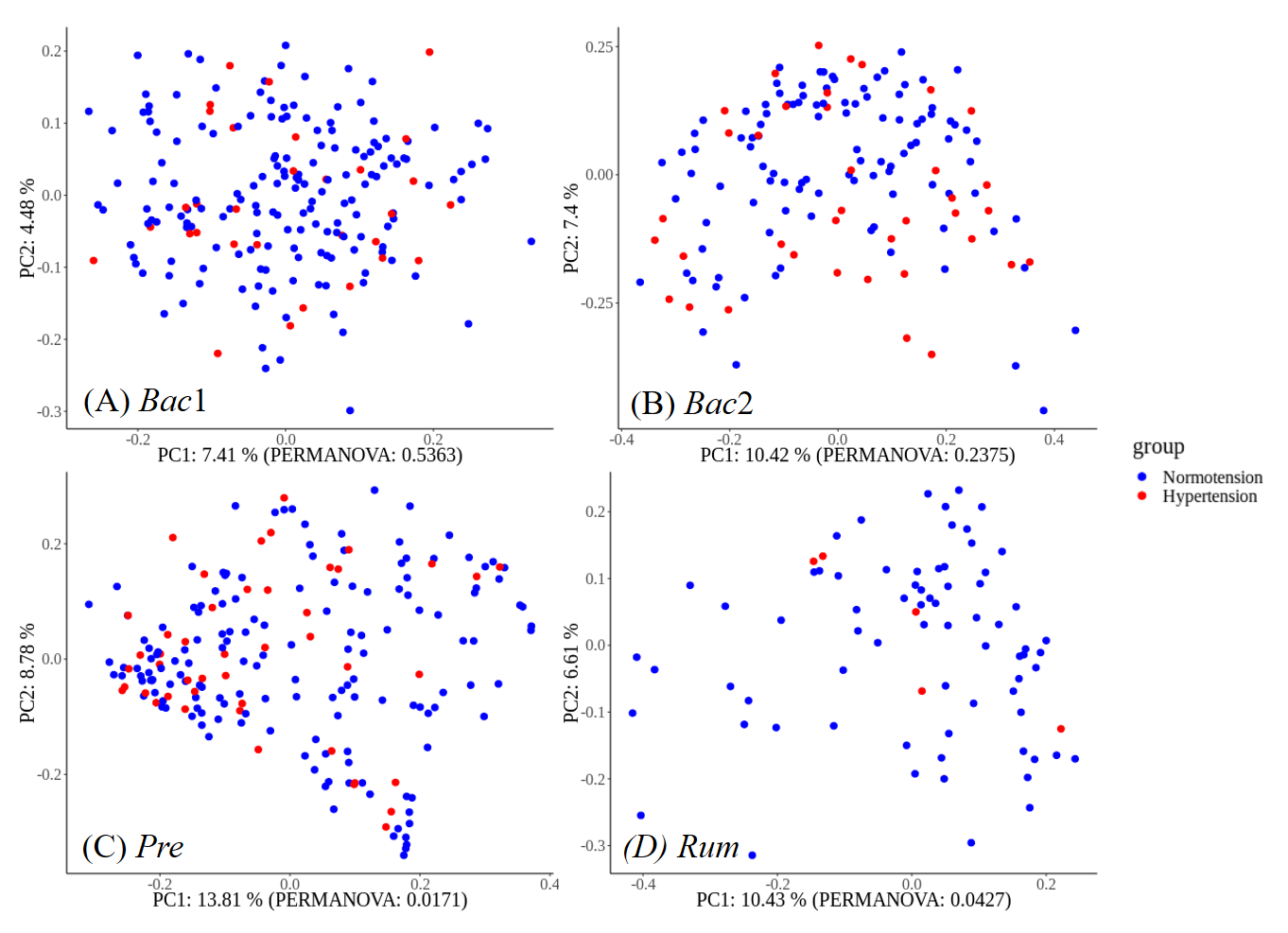

Supplement: Supplementary Figure 2 — Beta diversity as a principal coordinates analysis (PCoA) plot derived from Bray–Curtis distances of two blood pressure groups in each enterotype. There was no significant difference between the two groups for all four enterotypes. (A) Bac1 enterotype (B) Bac2 enterotype (C) Pre enterotype (D) Rum enterotype [file Image_2.tif]
